# Efficacy and tolerability of full spectrum hemp oil in dogs living with pain in common household settings

**DOI:** 10.3389/fvets.2024.1384168

**Published:** 2024-07-12

**Authors:** Chinmayee Panda, Thirumurugan Rathinasabapathy, Brandon Metzger, Sheila Dodson, Dirk Hanson, Jody Griffiths, Slavko Komarnytsky

**Affiliations:** ^1^Nutrition Innovation Center, Standard Process, Kannapolis, NC, United States; ^2^Plants for Human Health Institute, NC State University, Kannapolis, NC, United States; ^3^Animal Health Clinical Studies, Lenexa, KS, United States; ^4^Department of Food, Bioprocessing, and Nutrition Sciences, North Carolina State University, Raleigh, NC, United States

**Keywords:** whole food, botanical, dietary supplement, bioactive components, inflammation, hemp oil, pain management, nutrition

## Abstract

Lameness and restricted mobility are a significant concern in companion animals experiencing chronic pain, inflammation, or age-related pathologies. The growing awareness of health risks and side effects associated with the long-term use of prescription analgesics requires different management strategies to address these issues. In this study, we conducted a crossover evaluation of the effect of full spectrum hemp oil dosed orally at 2 mg/kg BID phytocannabinoids for 8 weeks in dogs (*n* = 37) living with pain in common household settings. Owner-reported canine pain, home activity, accelerometer-based activity, walkway-based gait, and tolerability were assessed at each phase of the study. Secondary endpoints included changes in blood biochemistry, liver enzymes, inflammatory biomarkers, and plasma metabolites. The intervention was positively associated with a decrease in pain scores (−46.2%, *p* = 0.0016), increased ability to walk up and down the stairs (10.6–14.7%, *p* < 0.05), and improved daily activity (25.9%, *p* = 0.0038). Decreases in plasma levels of proinflammatory cytokines TNF-α, IL-6, and IL-8 were also observed. Taken together, these findings suggest that the benefits of nutritional supplementation with hemp oil could include control of pain, greater mobility, and an overall improvement in the animal wellbeing.

## 1 Introduction

Pet owners and veterinary professionals prioritize the well-being of companion animals. Dogs commonly experience inflammation due to various factors like osteoarthritis, injuries, chronic immune disorders, or as a part of the natural aging process. Many of these health conditions are associated with pain and decreased quality of life, and their chronic nature implies that they cannot be cured efficiently (1). Risk factors associated with chronic inflammation and pain include both genetic factors such as age and breeding pedigree, as well as modifiable lifestyle factors including diet, physical activity, and body weight (2). Moreover, prolonged activation of local and systemic inflammatory responses induces sickness behavior and maladapted motivational responses (3). These pathologies are driven in part by the core factor of exaggerated T-cell effector and pro-inflammatory cytokine production responses that may include tumor necrosis factor alpha (TNF-α), interleukins IL-1β, IL-6, IL-17, IL-18, and messengers of prolonged immune activation such as nuclear factor kappa B (NF-κB), prostaglandin-endoperoxide synthase 2 (COX-2), and inducible nitric oxide synthase (iNOS) (4).

Mild and moderate acute pain and inflammation associated with these conditions can be managed short-term with analgesic drugs such as nonsteroidal anti-inflammatory drugs (NSAIDs). Despite their effectiveness, there is a need for additional alternatives due to potential limitations associated with long-term use (5). For example, dietary supplementation with omega-3 fatty acids (3.5% of total food intake) was shown to significantly improve some clinical outcomes and weight bearing in these settings (6).

The endocannabinoid system (ECS) is a physiological network of endogenous cannabinoids (2-arachidonoylglycerol and anandamide, among others) derived from the omega-3 and omega-6 metabolic pathways similar to other lipid mediators that ensure long-term regulation and balance of neural, metabolic, and immune functions (7). Endocannabinoids act as the retrograde signaling molecules at the classical cannabinoid receptors (CB1R, CB2R), as well as in a variety of the heteromer signaling networks mediated by the activity of the serotonin 5HT1A, dopamine D2R, vanilloid TRPV1, putative GPR55, and nuclear PPAR-γ receptors (8). External cannabinoids, such as those present in the *Cannabis sativa* L. plants and botanical formulations that contain hemp oils, have the capacity to interact with the same molecular targets and elicit a spectrum of physiological responses similar to endocannabinoids (9). The principal non-psychoactive cannabinoid cannabidiol (CBD) found in hemp oils has been well tolerated at a dose of 4 mg/kg for 6 months, and has shown promise in alleviating discomfort and inflammation in dogs (10). Its absorption and metabolism is greatly defined by dosage forms, routes of administration, and the fasting status of the subject (11). As such, different product formulations are expected to produce distinctive CBD kinetic profiles and tolerability outcomes (12).

Targeting the ECS with dietary interventions hold the potential for safe and effective management of various canine health conditions that cause chronic pain and inflammation, thus limiting dogs mobility and activity levels (13). Full spectrum hemp oils, alone or in combination with other omega-3 rich oils (14), provide a comprehensive approach to address these issues, but their potential is underexplored. In this study, we examined 8-week nutritional supplementation with full spectrum hemp oil standardized to contain 15 mg/mL phytocannabinoids on clinical measures of pain, pain severity, and activity in client-owned dogs attending veterinary offices for routine pain support. Secondary outcomes were to determine the tolerability of the intervention by assessing adverse effects, blood biochemistry panels, inflammatory biomarkers and plasma CBD levels.

## 2 Materials and methods

### 2.1 Animals and study protocol

The study population consisted of privately owned dogs presenting to Mariposa Veterinary Wellness Center (Lenexa, Kansas) during August–December 2022, treated as routine patients for physical evaluation and dietary supplementation for pain support. Owners completed a questionnaire to define the health history, type, and duration of analgesic or other medications taken. Animals were considered for inclusion in the study if they were (1) dogs of any breed or size, (2) 3–13 years of age, (3) had no recent use of hemp oils or CBD oil products in the last 60 days, and (4) received an affirmative confirmation of living with pain according to assessment by their owners, as well as by a veterinarian through the physical exam. Dogs were excluded from the study if (1) they exhibited evidence of uncontrolled renal, endocrine, neurologic, or neoplastic disease, (2) experienced changes in other medications, NSAIDS, or supplements during the study, or (3) failed the study procedures.

**Figure 1:**
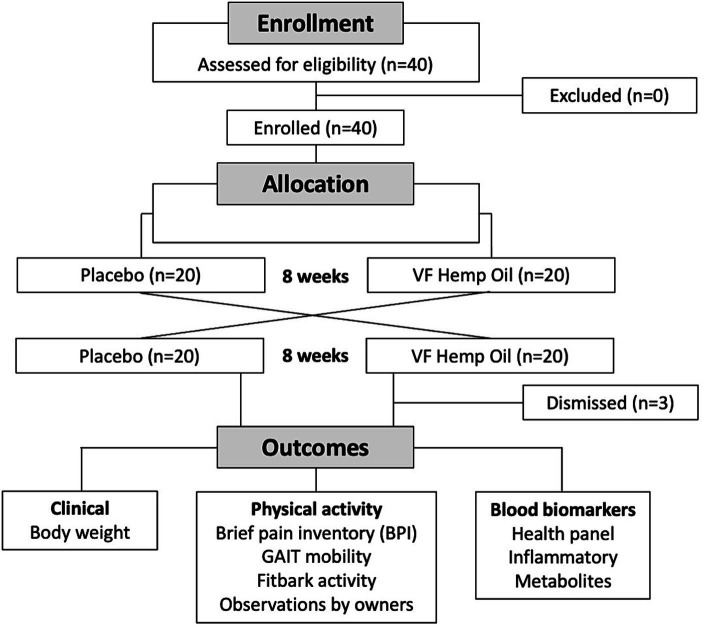
Flowchart of the study.

Flow of the animals through the study is shown in Figure 1. The study was a 16-week evaluation of privately owned dogs in either placebo or hemp oil phase, each lasting 8 weeks. The lack of washout between the phases is a procedural limitation of this study. The study followed a double-blind design as both dog owners and the participating veterinarians were masked from knowing which study treatment was received. Following provision of informed owner consent, 40 dogs were assigned 1:1 to either phase. No study participants had any exclusion criteria. Three intervention participants were later excluded from the study due to failure to comply with the study procedures after the first visit. At the time of study initiation (week 1, baseline), at the end of the first phase (week 8), and at the end of the second phase (week 16), each dog was evaluated by the study veterinarian and subjected to health blood panel as a part of the routine healthcare visit.

The study protocol was reviewed and approved by the Animal Health Clinical Studies (Lenexa, KS) and conducted by the attending veterinarians (SD and DH). The client-owned animals were treated as routine patients (15). Dog owners signed an informed client consent and were fully informed about any risks associated with the procedures.

### 2.2 Study intervention

The whole-food intervention was supplied as 1 fl oz (30 mL) VF Hemp Oil bottles by the manufacturer (Standard Process Inc., Palmyra, WI). The serving size was defined as 1 mL and included a blend of 35 mg hemp oil standardized to contain 15 mg total cannabinoids and 880 mg of organic extra virgin olive oil (carrier oil). The placebo was formulated to contain 915 mg of the carrier oil only. The nutritional intervention or placebo were administered by the owners orally or on top of the food, at the dose of 2 mg phytocannabinoids per kg body weight, twice daily for 8 weeks of each study phase.

### 2.3 Clinical observations, tolerance, and activity in home settings

Age, gender, breed, health and medication history were taken by the study staff at the study baseline. Body weight was measured using a calibrated scale. The Observational questionnaire was administered to all owners for recording of appetite, dietary changes, gastrointestinal symptoms (vomiting, diarrhea), sleeping patterns, physical ability to jump up and down the furniture, climb up and down the stairs, play quality, and overall wellbeing to monitor adherence and health-related events during the study. The appetite was scored on a 3-point scale as decreased (1), no change (2), or increased (3). The incidence of vomiting and diarrhea was reported as absent (0) or present (1) on a weekly basis. Sleep, play, and overall wellbeing scores were reported as worsened a lot (1), worsened a little (2), no change (3), improved a little (4), and improved a lot (5) scale. All questionnaires were administered 4 times, at weeks 1, 8, 9, and 16 of the study.

### 2.4 Owner-reported pain and pain severity

Owners were asked to rate the perceived pain in dogs in the last 7 days on a scale from 0 to 10, where 0 equals “no pain” and 10 is the “extreme pain.” The owners were also asked to self-report the perceived level of pain severity on dog’s body function including general activity, enjoyment of life, ability to rise, walk, run, climb, and overall quality of life using the Canine Brief Pain Inventory (CBPI) questionnaire.

### 2.5 Physical activity

Each dog was equipped with a flat collar with a FitBark (Kansas City, MO) accelerometer monitor attached (16). The FitBark monitor was located ventrally to ensure a snug fit with a two-finger gap between the collar and neck (17). Daily activity levels were recorded continuously for the duration of the study, and downloaded for further analysis in the form of activity counts generated from 3D accelerometer readings.

### 2.6 Gait assessment

Dog gait analysis for identifying lameness and tracking progress was performed using the GAIT4Dog Gait Analysis System (CIR Systems, Franklin, NJ) 4 times, at weeks 1, 8, 9, and 16 of the study. The gait parameters were calculated automatically and recorded as step/stride ratio (gait symmetry, expected at 50%), stance time % (the weight bearing portion of the gate cycle), gait lameness score (expected at 100% for a healthy animal), hind reach (a line of progression from the heel center of the hind paw to the heel center of the previous fore paw on the same side), and overall gait (18).

### 2.7 Blood panel

Blood samples were obtained at baseline (week 1) and the end of each phase (weeks 8 and 16). The samples were collected into BD Vacutainer EDTA tubes for complete blood count (CBC), and BD Vacutainer Lithium Heparin Plasma Separator tubes for electrolytes, metabolic, lipid, and hepatic panels. All blood biochemistry tests were performed at the IDEXX Reference Laboratories (Westbrook, ME).

### 2.8 Inflammatory markers

Plasma TNF-α, interleukins IL-1β, IL-6, IL-8, and IL-10 were quantified using the respective canine Invitrogen ELISA kits (ThermoFisher, Waltham, MA). Blood samples were collected in BD Vacutainer K2 EDTA tubes and processed according to the manufacturer’s instructions. Aliquots of plasma were stored at −80°C until analysis.

### 2.9 Plasma CBD levels

All chemicals were purchased from Sigma (Saint Louis, MO) unless specified otherwise. All solvents were of HPLC grade and purchased from VWR (Radnor, PA). Plasma samples were analyzed using a Shimadzu Prominence LC-2030C HPLC system equipped with a pump (LC-20AT), an autosampler (SIL-20A), a diode array detector (SPD-M20A) and an automatic column temperature control oven (CTO-20A). Briefly, plasma samples were extracted as described previously (7). Internal analytical standard was 4,4-dichlorodiphenyl-trichloroethane (DDT) at 50 μg/mL.

Separation was performed on Restek Ultra C18 column (250 × 4.6 mm, 5 μ) at a column temperature of 30°C. The binary mobile phase consisted of 0.1% formic acid in water (Eluent A) and 100% acetonitrile (Eluent B). Each run was followed by an equilibration time of 10 min. Ultraviolet (UV) spectra were monitored at 220 nm using the flow rate of 1 mL/min and injection volume of 20 μL. The data were collected and analyzed with LC solution (Shimadzu, Nakagyoku, Kyoto, Japan) software. Peaks were identified based on comparison of retention times and UV spectra with those of authentic cannabinoid standards (Shimadzu #220-91239-21). The assay was linear in the range of 10–10,000 ng/mL and LOQ of 10 ng/mL similar to a previously published method (19).

### 2.10 Statistics

Statistical analyses were performed using Prism 8.0 (GraphPad Software, San Diego, CA). All statistical analyses were conducted on an intention-to-treat basis and values were expressed as means ± standard deviations (SD), unless specified otherwise. Descriptive statistics and unpaired two-tailed t-test was used to evaluate differences between the two study groups. Repeated measures two-way ANOVA with Sidak multiple comparisons test was used to evaluate significant interactions. Statistical significance was set as ^*^p < 0.05, ^**^p < 0.01, and ^***^p < 0.001.

## 3 Results

### 3.1 Study participants

A total of 40 dogs were selected to start in the placebo-hemp oil (*n* = 20) or hemp oil-placebo phase of the crossover evaluation (*n* = 20; Figure 1). At the baseline, the aggregated average weight and pain scores of the study groups remained within 1 standard deviation (Table 1). As per study protocol, 37 dogs completed the study, and 3 dogs were excluded from the study due to failure to comply with the study procedures after the first visit. At baseline (week 1), aggregated average weight and pain scores were similar for both groups (Table 1). Due to the crossover nature of the study, all dogs that completed both phases of the study served as their own controls, thus minimizing the risks of the confounding effects.

**Table 1:** Clinical data at baseline (±SD).

Measures	Started on placebo (*n* = 20)	Started on hemp oil (*n* = 20)
Age (years)	8.7 ± 3.1	9.4 ± 2.4
Gender (female/male, %)	14/6 (70.0%)	13/7 (65.0%)
Weight, kg	21.94 ± 9.28	21.73 ± 10.59
Canine BPI score, points	21.08 ± 4.79	25.44 ± 4.89

BPI, Brief Pain Inventory.

### 3.2 Pain severity

**Figure 2:**
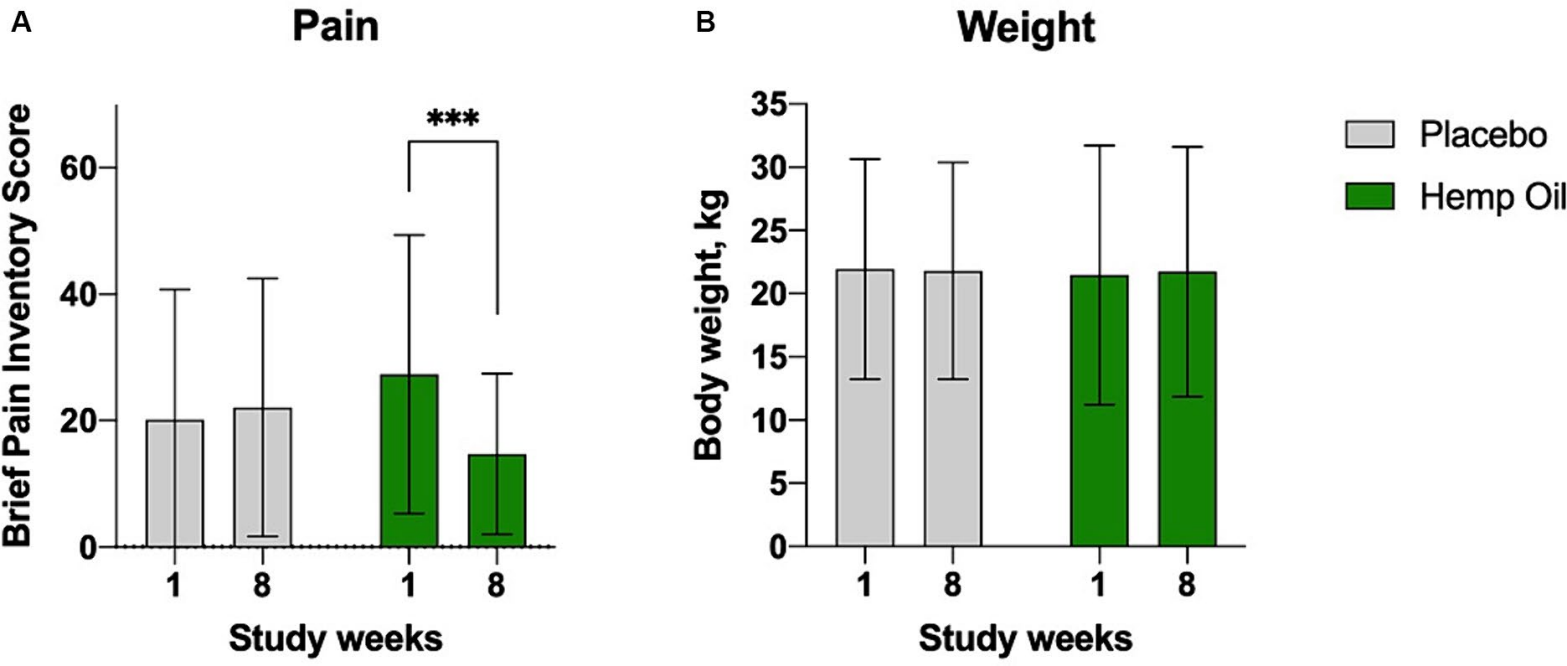
Average canine brief pain inventory (CBPI) scores **(A)** reported at baseline (week 1) and the end of each crossover phase (week 8). **(B)** These effects were observed despite no changes in dogs’ body weight. Results were expressed as means ± SD (*n* = 37). Data were analyzed by two-way ANOVA corrected for multiple comparisons with a Sidak test (^***^p < 0.001).

For the primary outcome measure, the mean (± SD) change from baseline in average owner-reported pain scores at the end of the 8-week intervention phase was greatest in the supplemented cohort (−12.6 canine BPI points, *p* = 0.0016; Figure 2A). The placebo group has not experienced significant changes in the perceived pain scores. This effect was observed in the absence of any changes in dogs body weight (Figure 2B). The mean overall difference in the BPI score changes of the hemp oil-treated animals relative to placebo was −14.6 ± 4.8 BPI points (95% CI: −4.8 to −24.3, *p* = 0.0045).

### 3.3 Tolerance assessment

**Figure 3:**
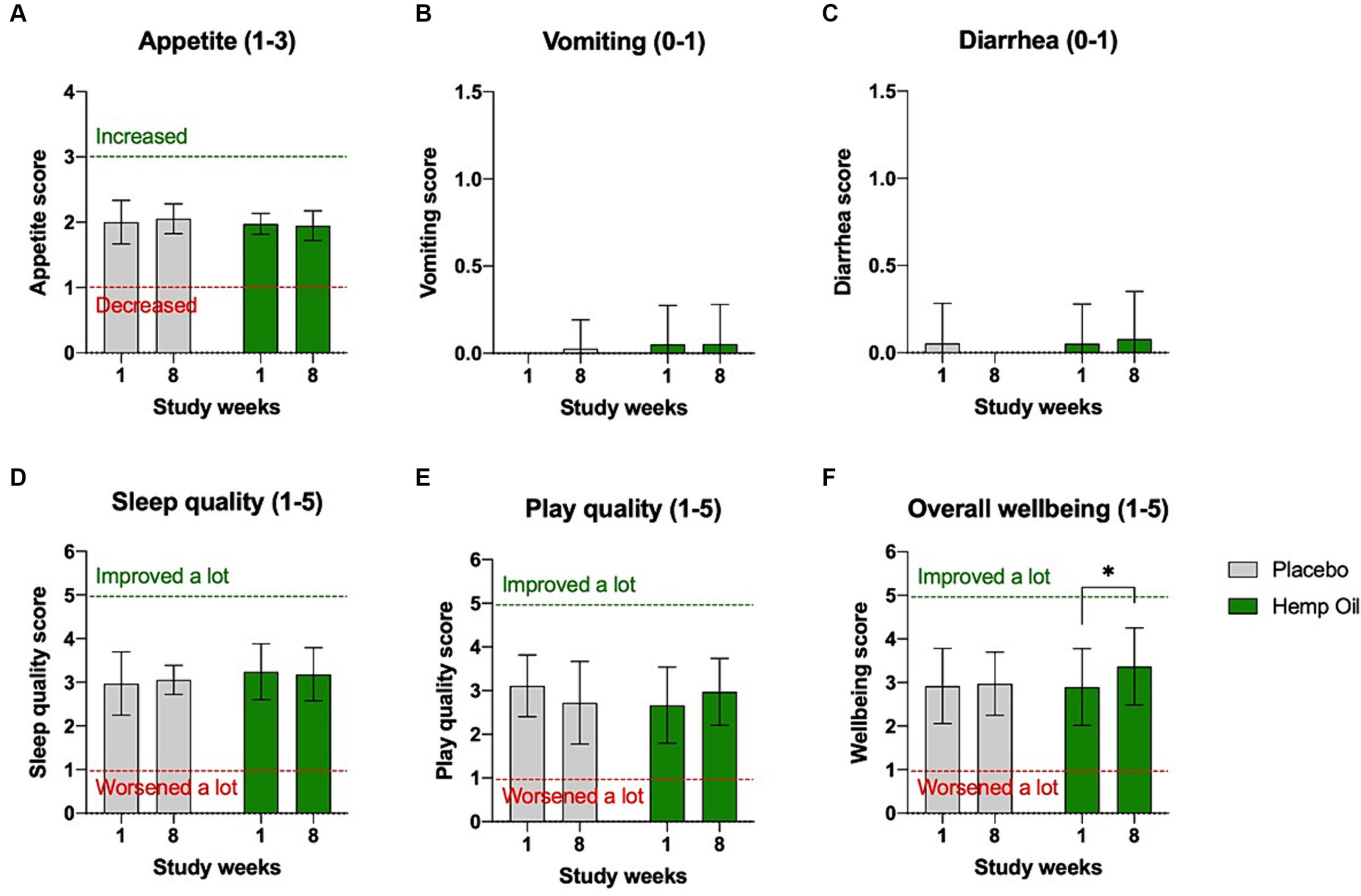
Owner-reported tolerance of the investigational product. No significant changes in **(A)** appetite, **(B)** vomiting, **(C)** diarrhea, **(D)** sleep quality, and **(E)** play quality were reported in either intervention or placebo phases of the study. **(F)** A moderate increase in the overall wellbeing score was observed in the intervention cohort. Results were expressed as means ± SD (*n* = 37). Data were analyzed by two-way ANOVA corrected for multiple comparisons with a Sidak test (^*^p < 0.05).

No major adverse effects attributable to the intervention were reported during the study. There were no changes in dogs’ appetite as reported by the owners. The incidence of vomiting or diarrhea were rare and equally distributed between the intervention and placebo groups. No significant changes in sleep or play quality were observed. The owners reported a moderate improvement in the perceived overall wellbeing of the supplemented group, from the average score of 2.9 (no change) during the study week 1 to 3.4 (improved a little) during the study week 8 (*p* = 0.03; Figures 3A–F).

Laboratory safety endpoints included data from the complete blood count (CBC), electrolytes, metabolic, lipid, and hepatic panels. This data showed no significant changes in the majority of the parameters tested (Supplementary Table S1). Minor changes included decreased basophils (within a normal reference interval that includes zero basophils), a small decrease in blood urea nitrogen (within a normal reference range of 7–26 mg/dL), and a mildly elevated alkaline phosphatase (normal reference range of 7–115 U/L).

### 3.4 Activity in home settings

**Figure 4:**
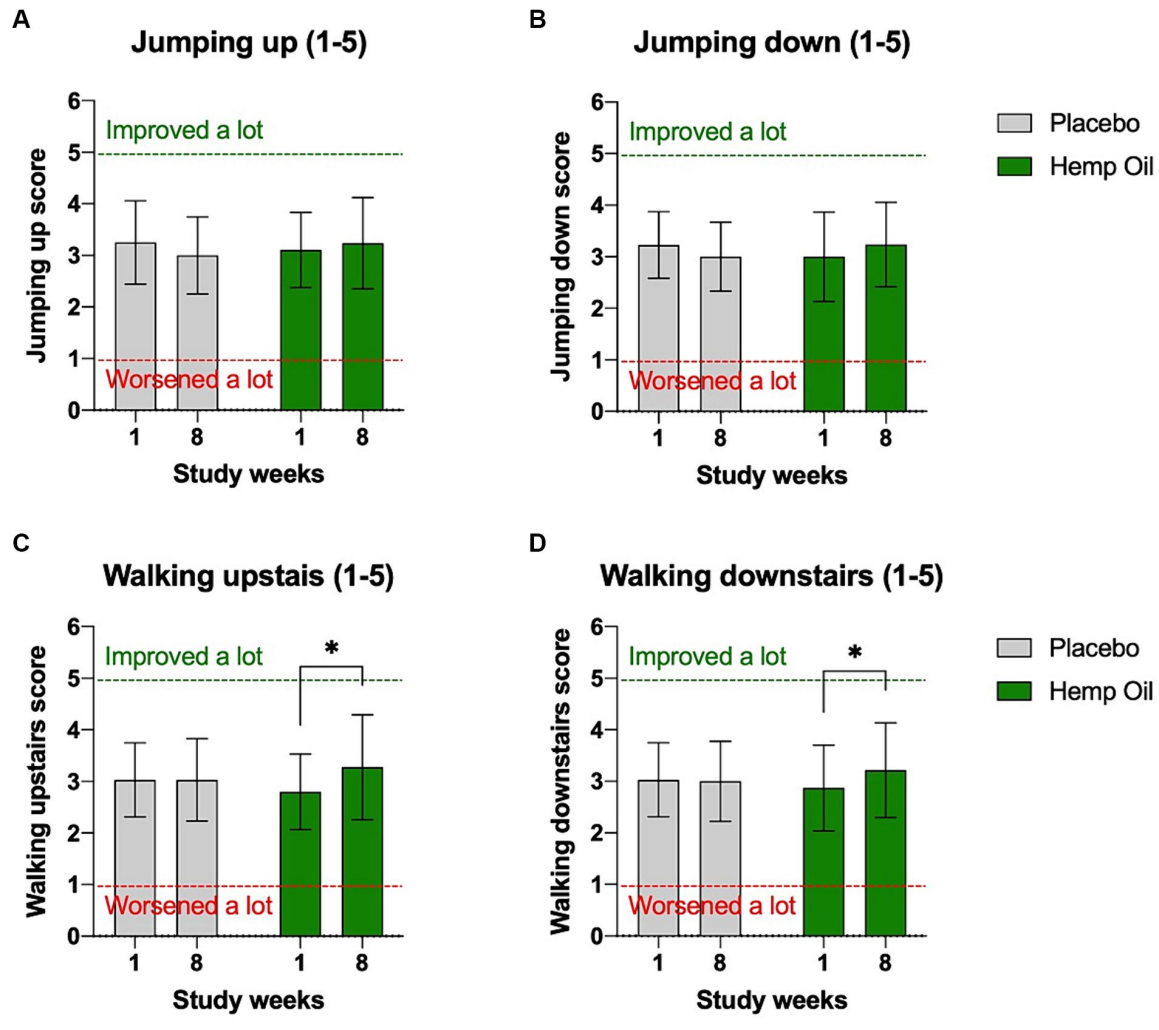
Owner-reported physical abilities of dogs in the home settings. Average score included dogs’ ability to **(A)** jump on the furniture, **(B)** jump off the furniture, **(C)** climb up the stairs, and **(D)** climb down the stairs. Results were expressed as means ± SD (*n* = 37). Data were analyzed by two-way ANOVA corrected for multiple comparisons with a Sidak test (^*^p < 0.05).

Owners were instructed to observe and record a range of physical activities at home as a part of the weekly observational questionnaire. These included dog’s ability to jump up and down from the furniture, as well as walk up and down the stairs. Hemp oil supplementation moderately improved dogs’ performance on the stairs in both directions. Their ability to climb upstairs improved from the average score of 2.8 to 3.3 (*p* = 0.011), while the climbing downstairs improved from the average score of 2.9 to 3.2 (*p* = 0.041). Placebo cohort showed no significant changes in physical activity in home settings (Figures 4A–D). The mean overall difference in improved performance on stairs of the hemp oil-treated animals relative to placebo was 0.53 ± 0.26 points (95% CI: 0.01 to 1.05, *p* = 0.047) when walking up, and 0.39 ± 0.24 points (95% CI: 0.09 to 0.87, *p* = 0.049) when walking down.

### 3.5 FitBark activity

**Figure 5:**
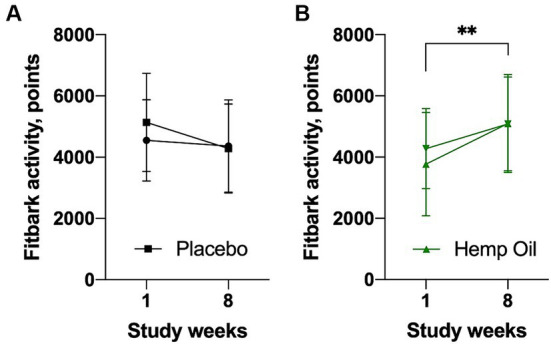
Average daily physical activity of the study dogs as recorded by the FitBark accelerometer (FitBark points) during the **(A)** placebo and **(B)** hemp oil intervention phases of the study. Results were expressed as means ± SD (*n* = 37). Data were analyzed by two-way ANOVA corrected for multiple comparisons with a Sidak test (^**^p < 0.01).

FitBark activity values of both cohorts at the beginning of the study phase (week 1) and the end of the study phase (week 8) are shown in Figure 5. No significant changes in physical activity were observed during the placebo phase. Hemp oil supplementation was associated with a marked increase in FitBark activity, from the average of 3,771 FitBark points during week 1 to 5,083 FitBark points during week 8 of the intervention study phase (an increase of 34.8%, *p* = 0.0038; Figures 5A,B). The mean overall increase in Fitbark activity of the hemp oil-treated animals relative to placebo was 1,627 ± 203 points (95% CI: 1,216 to 2,038, *p* < 0.0001).

### 3.6 Gait analysis

**Figure 6:**
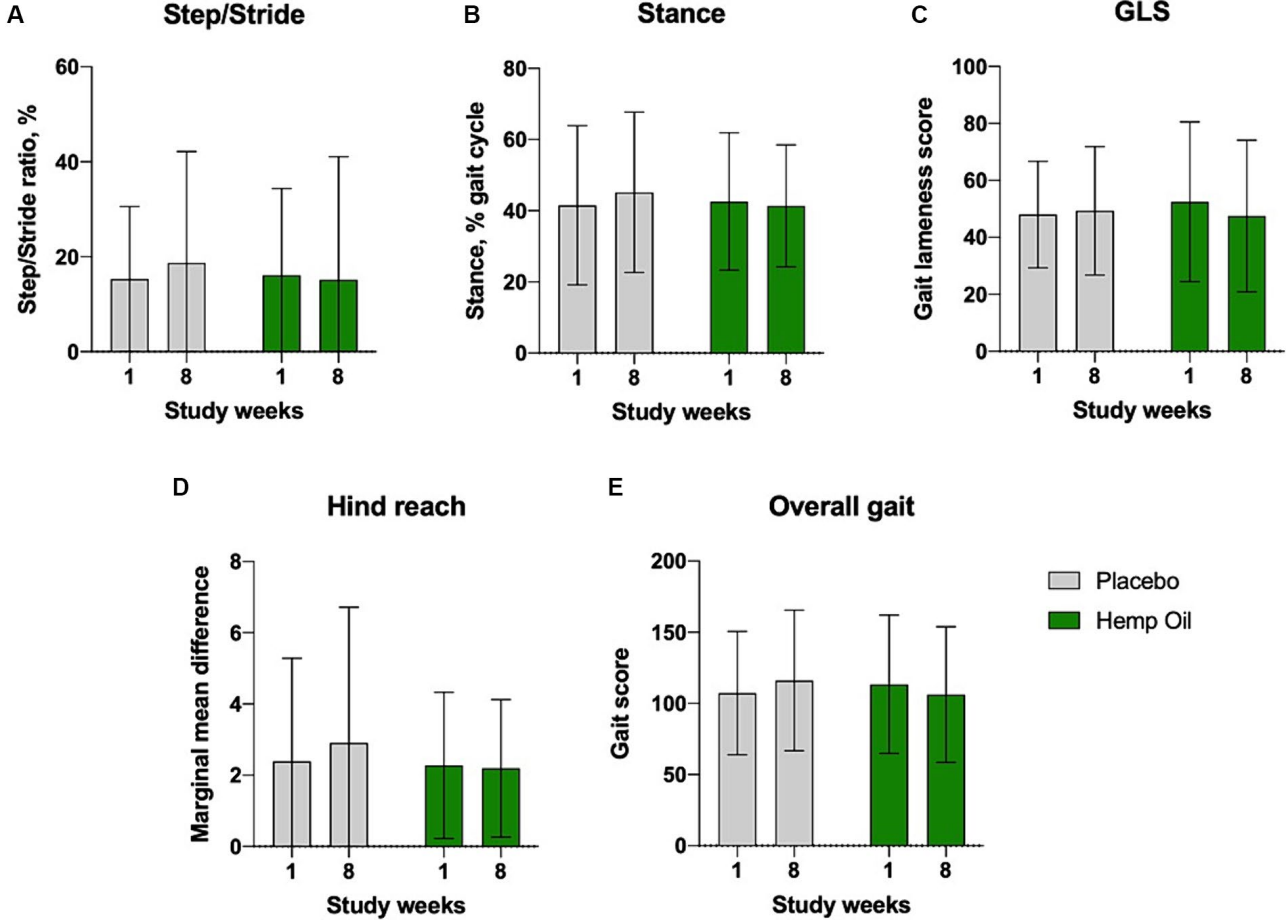
Average gait lameness scores of the study dogs as recorded by the GAIT4 walkway system that automatically calculated the parameters of **(A)** gait symmetry, **(B)** gait cycle, **(C)** GLS, gait lameness score, **(D)** hind reach, and **(E)** overall gait. Results were expressed as means ± SD (*n* = 37). Data were analyzed by two-way ANOVA corrected for multiple comparisons with a Sidak test.

Dogs’ overall limb health was evaluated using the GAIT4 walkway system that automatically recorded and calculated each limb functional parameters and transformed them into the average scores for gait symmetry (step/stride ratio), gait cycle (stance time), gait lameness, hind reach, and overall gait. The mean overall change in gait score of the hemp oil-treated animals relative to placebo did not reach significance and was observed at 22.3 ± 12.9 points (95% CI: −4.1 to 48.7, *p* < 0.095; Figure 6).

### 3.7 Inflammatory biomarkers

**Figure 7:**
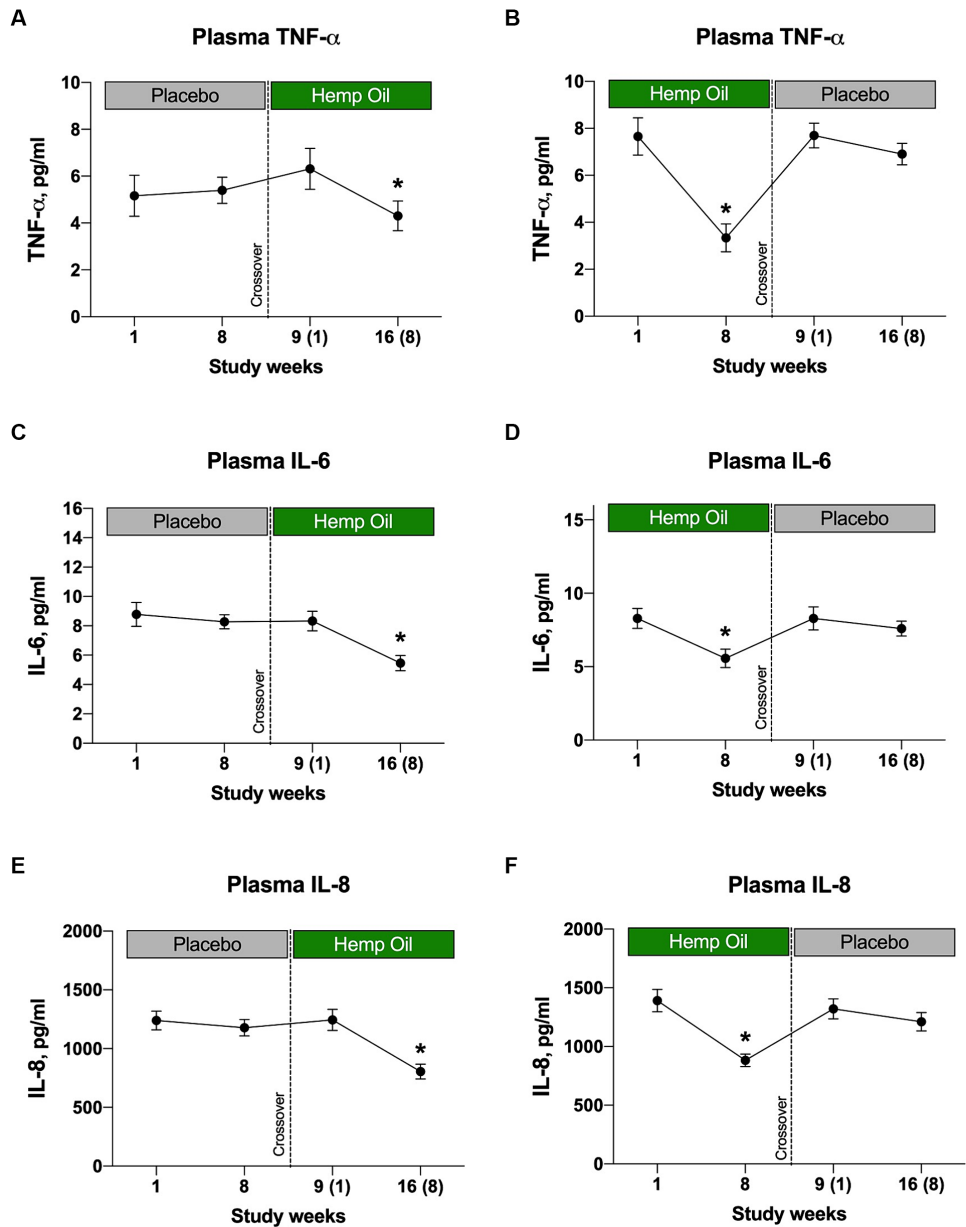
Hemp oil intervention reduces plasma inflammatory cytokine levels of **(A, B)** TNF-α, **(C, D)** IL-6, and **(E, F)** IL-8. Results were expressed as means ± SEM (*n* = 37). Data were analyzed by repeated measures two-way ANOVA corrected for multiple comparisons with a Sidak test (^*^p < 0.05).

Hemp oil supplementation decreased plasma levels of the three pro-inflammatory biomarkers, TNF-α, IL-6, and IL-8 (Figure 7), while having no effect on interleukins IL-1β and IL-10 (data not shown). The decrease was evident from either sequence of the crossover study, as both placebo-to-hemp oil (Figures 7A,C,E), as well as hemp oil-to-placebo (Figures 7B,D,F) cohorts exhibited reduced plasma cytokines only at the hemp oil phase of the study. The cytokine-lowering effects were transitory and disappeared once hemp oil supplementation ended in the respective phase. Largest plasma cytokine decreases were observed for TNF-α (−31.9% and −43.5%, *p* < 0.05) and IL-6 (−34.6% and −32.7%, *p* < 0.05; Figures 7A–D).

### 3.8 Plasma CBD levels

**Figure 8:**
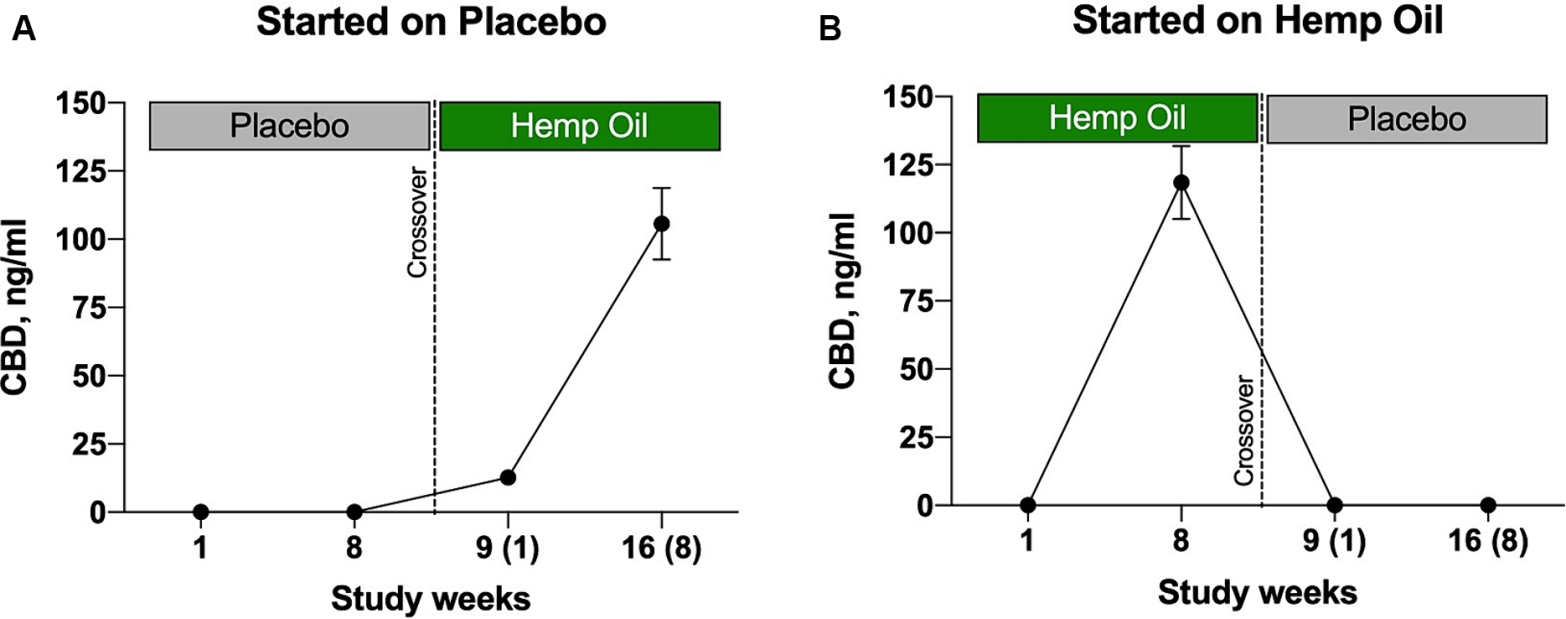
Levels of hemp oil plasma metabolite CBD in the study participants undergoing **(A)** placebo-to-hemp oil sequence and **(B)** hemp oil-to-placebo sequence of the crossover study. Results were expressed as means ± SD (*n* = 37). Data were analyzed by repeated measures two-way ANOVA corrected for multiple comparisons with a Sidak test.

After 8 weeks of intervention with hemp oil dosed at 2 mg/kg total phytocannabinoids, a significant increase of CBD was observed in plasma of the animals in the hemp oil phase of the study, but not placebo (Figure 8). It can be noted that CBD peaked in plasma at 105.6–118.4 ng/mL (Figures 8A,B). These findings indicated that CBD reached bloodstream at the physiologically relevant concentrations under the current study duration and dosing regimen.

## 4 Discussion

Dogs, much like humans, experience discomfort and limited mobility when they suffer from conditions such as arthritis, hip dysplasia, or other chronic inflammatory disorders (20). The persistent pain can lead to behavioral changes, including irritability, restlessness, and aggression. Dogs may become less active, reluctant to play or exercise, and may have difficulty climbing stairs or getting in and out of vehicles (21). This reduced activity level can contribute to obesity, which in turn exacerbates joint pain and inflammation, creating a vicious cycle of discomfort (22). It is essential for pet owners to recognize the signs of chronic inflammation and pain in their dogs, and seek appropriate veterinary care and treatments to improve their quality of life.

In this study, we demonstrated that the 8-week use of a whole-food nutritional supplement containing full spectrum hemp oil standardized to 15 mg phytocannabinoids (oral dosing of 2 mg/kg body weight BID), decreased owner-reported pain in dogs. The greatest effects were observed within the intervention group in the areas of CBPI scores (−46.2% reduction), ability to walk up and down the stairs (14.7% and 10.6% increases, respectively), and overall daily activity (an increase of 25.9%), while no changes were noted for dogs receiving a placebo treatment. These differences were also observed between the treated and placebo animals, while the changes in gait analysis remained non-significant both within and in between the study groups. There was a non-significant trend in observing lower cumulative deviation scores (decreased lameness, a more optimal functionality of the study participants limbs) that was most prominent in gate lameness [GLS, a change from the average score of 52.5 during week 1 to 47.5 during week 8 of the intervention study phase (a decrease of 9.5%, *p* = 0.307)]. This partially contrasts to a previous study that showed no difference between groups in activity and gait measures in osteoarthritic dogs receiving 2.5 mg/kg CBD oil for 6 weeks (23), but agrees with the findings of a repeated-dose 28-day study that used 2 mg/kg CBD oil and reported a significant decrease in the CBPI scores and increased dog activity (11). We also observed a mild increase in serum alkaline phosphatase reported in the latter study (11). Another study that investigated oral transmucosal delivery of CBD oil at the same target dose of 2 mg/kg for 12 weeks also reported significantly lower CBPI scores (24). Taken together, these findings suggest that the benefits of nutritional supplementation with hemp oils containing CBD could include better control of pain, greater mobility, and an overall improvement in the animal wellbeing.

CBD absorption and metabolism is different between dogs and humans, both in concentrations of free state CBD, as well as in accumulation of the major glucuronidated metabolites in urine (7-hydroxy-CBD vs. 6-hydroxy-CBD) (25). Long-term daily feeding of 4 mg/kg CBD for 6 months seems to be well-tolerated by healthy dogs (10), and plasma CBD concentrations typically peak in the range from 10 ng/mL (26) to 300 ng/mL (27), which is comparable to findings observed here. The hemp oil was well accepted in this study with only rare incidence of vomiting or diarrhea, and body weight, appetite, or sleep were not affected at the dose regimen used. Similar results were reported for healthy dogs and cats dosed with CBD hemp oils at 2 mg/kg for 12 weeks (27), or CBD/CBDA hemp oil at the same dosage and duration (28). Our study also showed concurrent increases in plasma CBD and decreases in 3 out of 5 tested plasma inflammatory cytokines (TNF-α, IL-6, and IL-8) only in the active phase of the crossover evaluation, suggesting a possible molecular mechanism behind the observed beneficial physiological effects (29).

While this study offers novel insights into a whole-food, nutritional intervention and its potential application to managing pain and mobility in dogs, it has several strengths and limitations. Major strengths include a crossover evaluation that uses the companion dogs living in common households and covers a variety of ages, breeds, and body weights. However, due to the natural variability of cannabinoid metabolites in hemp oils of different geographic, processing, and manufacturing origin (30), findings with this particular intervention cannot be used to support the overall safety of all hemp oils in dogs.

## 5 Conclusion

Our work highlights the feasibility of translating the whole-food hemp oil intervention capable of supporting the animal chronic pain and immune health networks. The response to supplementation was sustained for the duration of the active phases of the study, and was associated with measurable improvements in pain and mobility levels. Therefore, full spectrum hemp oil dosed at 2 mg/kg BID may benefit dogs that have inadequate chronic pain management or those suffering from the systematic side effects of pain medications.

## Data availability statement

The original contributions presented in the study are included in the article/Supplementary material, further inquiries can be directed to the corresponding author.

## Ethics statement

The study protocol was reviewed and approved by the Animal Health Clinical Studies (Lenexa, KS) and conducted to the highest standard of veterinary care by the attending veterinarians (SD and DH). The client-owned animals were treated as routine patients. Written informed consent was obtained from the owners for the participation of their animals in this study.

## Author contributions

CP: Conceptualization, Data curation, Formal analysis, Investigation, Methodology, Writing – original draft, Writing – review & editing. TR: Data curation, Investigation, Methodology, Writing – original draft, Writing – review & editing. BM: Conceptualization, Project administration, Resources, Supervision, Writing – original draft, Writing – review & editing. SD: Conceptualization, Data curation, Formal analysis, Investigation, Methodology, Project administration, Resources, Supervision, Writing – original draft, Writing – review & editing. DH: Conceptualization, Data curation, Formal analysis, Investigation, Methodology, Project administration, Resources, Supervision, Writing – original draft, Writing – review & editing. JG: Investigation, Project administration, Resources, Writing – original draft, Writing – review & editing. SK: Investigation, Supervision, Writing – original draft, Writing – review & editing.
