# Supplementary material for: Efficacy and tolerability of full spectrum hemp oil in dogs living with pain in common household settings
Source: Front Vet Sci. 2024 Jul 12;11:1384168. doi: 10.3389/fvets.2024.1384168 (PMC11272626; doi:10.3389/fvets.2024.1384168)
Supplement: Supplementary file 1 [file Table_1.DOCX]

Supplementary Material

**Panda et al.,** Efficacy and tolerability of full spectrum hemp oil in dogs living with pain in common household settings

**Supplementary Table 1.** CBC, electrolytes, metabolic, lipid, and hepatic panels data (±SD).

| **Blood panel parameters** | **Baseline** | **Study groups** | |  |
| --- | --- | --- | --- | --- |
|  |  | **Placebo** | **Hemp Oil** | **P^1^** |
| RBC, millions/μl | 7.86 ± 0.74 | 7.83 ± 0.78 | 7.84 ± 0.77 | 0.880 |
| Hematocrit, % | 52.43 ± 5.25 | 54.39 ± 5.54 | 54.53 ± 5.33 | 0.736 |
| Hemoglobin, g/dl | 19.01 ± 2.13 | 20.72 ± 10.52 | 19.14 ± 2.29 | 0.376 |
| MCV, fl | 66.82 ± 2.70 | 70.06 ± 4.61 | 69.59 ± 2.45 | 0.480 |
| MCH, pg | 24.16 ± 1.16 | 24.18 ± 1.19 | 24.31 ± 1.45 | 0.304 |
| MCHC, g/dl | 36.21 ± 0.90 | 34.80 ± 0.83 | 34.89 ± 1.15 | 0.519 |
| Reticulocytes, % | 0.79 ± 0.30 | 0.92 ± 0.38 | 0.89 ± 0.33 | 0.194 |
| Reticulocytes, thousands/μl | 62.21 ± 24.78 | 72.00 ± 30.61 | 68.21 ± 26.39 | 0.238 |
| Reticulocyte hemoglobin, pg | 26.20 ± 1.99 | 25.97 ± 1.46 | 26.19 ± 1.24 | 0.098 |
| WBC, thousands/μl | 7.95 ± 2.40 | 8.41 ± 3.76 | 7.98 ± 3.58 | 0.095 |
| Neutrophils, % | 44.99 ± 20.08 | 45.40 ± 22.17 | 46.66 ± 23.45 | 0.203 |
| Lymphocytes, % | 44.89 ± 21.07 | 45.19 ± 22.92 | 44.81 ± 23.16 | 0.682 |
| Monocytes, % | 3.21 ± 0.87 | 3.58 ± 0.96 | 3.43 ± 0.87 | 0.373 |
| Eosinophils, % | 6.74 ± 5.06 | 5.67 ± 3.90 | 4.99 ± 4.20 | 0.239 |
| Basophils, % | 0.16 ± 0.12 | 0.16 ± 0.10 | 0.12 ± 0.11 | 0.085 |
| Neutrophils, thousands/μl | 7.03 ± 11.22 | 5.71 ± 3.11 | 5.51 ± 3.24 | 0.410 |
| Lymphocytes, thousands/μl | 2.05 ± 0.77 | 1.94 ± 0.68 | 1.83 ± 0.66 | 0.125 |
| Monocytes, thousands/μl | 0.30 ± 0.31 | 0.31 ± 0.22 | 0.27 ± 0.11 | 0.168 |
| Eosinophils, thousands/μl | 0.53 ± 0.49 | 0.44 ± 0.35 | 0.38 ± 0.30 | 0.237 |
| Basophils, thousands/μl | 0.013 ± 0.011 | 0.012 ± 0.007 | 0.009 ± 0.008* | 0.029 |
| Platelets, thousands/μl | 303.2 ± 95.3 | 250.9 ± 99.9 | 251.9 ± 99.6 | 0.950 |
| Glucose, mg/dl | 90.36 ± 18.26 | 89.62 ± 12.71 | 90.71 ± 15.39 | 0.694 |
| SDMA, μg/dl | 12.87 ± 2.99 | 12.19 ± 3.36 | 12.06 ± 2.94 | 0.721 |
| Creatinine, mg/dl | 1.17 ± 0.39 | 1.14 ± 0.40 | 1.14 ± 0.42 | 0.929 |
| BUN, mg/dl | 20.06 ± 5.30 | 20.71 ± 6.22 | 19.29 ± 5.65* | 0.035 |
| BUN to Creatinine ratio | 18.97 ± 7.08 | 20.30 ± 8.30 | 18.85 ± 7.75 | 0.051 |
| Phosphorus, mg/dl | 3.75 ± 0.74 | 3.84 ± 0.60 | 3.62 ± 0.59 | 0.056 |
| Calcium, mg/dl | 10.20 ± 0.47 | 10.47 ± 0.53 | 10.40 ± 0.68 | 0.590 |
| Sodium, mmol/l | 148.8 ± 1.8 | 150.4 ± 2.0 | 150.5 ± 2.4 | 0.835 |
| Potassium, mmol/l | 4.54 ± 0.26 | 4.55 ± 0.28 | 4.49 ± 0.34 | 0.307 |
| Sodium to Potassium ratio | 32.91 ± 1.94 | 33.09 ± 2.29 | 32.81 ± 5.69 | 0.786 |
| Chloride, mmol/l | 112.8 ± 2.6 | 112.8 ± 2.5 | 113.0 ± 2.9 | 0.617 |
| Total CO_2_, mmol/l | 18.82 ± 1.75 | 19.76 ± 2.13 | 19.71 ± 2.21 | 0.897 |
| Anion gap, mmol/l | 21.79 ± 2.19 | 22.56 ± 3.01 | 22.29 ± 3.00 | 0.715 |
| Total protein, g/dl | 6.45 ± 0.49 | 6.71 ± 0.53 | 6.63 ± 0.53 | 0.229 |
| Albumin, g/dl | 3.29 ± 0.35 | 3.42 ± 0.37 | 3.42 ± 0.34 | 0.847 |
| Globulin, g/dl | 3.16 ± 0.45 | 3.29 ± 0.43 | 3.21 ± 0.41 | 0.061 |
| Albumin to Globulin ratio | 1.07 ± 0.20 | 1.06 ± 0.19 | 1.08 ± 0.19 | 0.160 |
| Alanine transaminase ALT, U/l | 49.26 ± 34.67 | 54.09 ± 45.26 | 54.79 ± 61.55 | 0.953 |
| Aspartate aminotransferase AST, U/l | 34.79 ± 25.24 | 32.50 ± 10.55 | 43.85 ± 67.18 | 0.331 |
| Alkaline phosphatase ALP, U/l | 78.9 ± 136.1 | 88.9 ± 148.0 | 164.2 ± 245.5 | 0.081 |
| Gamma-glutamyl transferase GGT, U/l | 1.75 ± 2.22 | 1.99 ± 2.02 | 1.83 ± 1.83 | 0.242 |
| Total bilirubin, mg/dl | 1.30 ± 1.48 | 1.95 ± 2.71 | 1.91 ± 2.34 | 0.923 |
| Cholesterol, mg/dl | 228.6 ± 72.2 | 241.3 ± 66.4 | 239.3 ± 88.6 | 0.874 |
| Amylase, U/l | 653.8 ± 247.7 | 666.8 ± 245.2 | 580.4 ± 233.1 | 0.055 |
| Lipase, U/l | 120.1 ± 149.1 | 119.5 ± 95.2 | 121.1 ± 165.4 | 0.949 |
| Creatine kinase CK, U/l | 249.5 ± 565.6 | 157.5 ± 95.2 | 151.8 ± 58.8 | 0.642 |
